# Evolving symbolic density functionals

**DOI:** 10.1126/sciadv.abq0279

**Published:** 2022-09-09

**Authors:** He Ma, Arunachalam Narayanaswamy, Patrick Riley, Li Li

**Affiliations:** ^1^ Google Research, Mountain View, CA 94043, USA.; ^2^ Relay Therapeutics, 399 Binney Street, 2nd Floor, Cambridge, MA 02139, USA.

## Abstract

Systematic development of accurate density functionals has been a decades-long challenge for scientists. Despite emerging applications of machine learning (ML) in approximating functionals, the resulting ML functionals usually contain more than tens of thousands of parameters, leading to a huge gap in the formulation with the conventional human-designed symbolic functionals. We propose a new framework, Symbolic Functional Evolutionary Search (SyFES), that automatically constructs accurate functionals in the symbolic form, which is more explainable to humans, cheaper to evaluate, and easier to integrate to existing codes than other ML functionals. We first show that, without prior knowledge, SyFES reconstructed a known functional from scratch. We then demonstrate that evolving from an existing functional ωB97M-V, SyFES found a new functional, GAS22 (Google Accelerated Science 22), that performs better for most of the molecular types in the test set of Main Group Chemistry Database (MGCDB84). Our framework opens a new direction in leveraging computing power for the systematic development of symbolic density functionals.

## Teaser

Machine learning composes density functionals in symbolic forms with similar simplicity as human-designed ones.

## INTRODUCTION

Quantum mechanical simulations of molecules and materials are playing an increasingly important role in chemistry, physics, and materials sciences. Density functional theory (DFT) (*1*) has been one of the most successful methods for determining electronic structures of molecules and materials from first principles (*2*, *3*) and has been widely used for the design and characterization of novel drugs (*4*), catalysts (*5*), and functional materials (*6*). Most DFT calculations performed today adopt the Kohn-Sham (KS) scheme (*7*). KS-DFT maps the challenging problem of solving the many-body Schrodinger equation of interacting electrons into the solution of one-body KS equations, with the complicated many-body effect treated with the exchange-correlation (XC) functional. This mapping is, in principle, exact. However, because the exact form of the XC functional is unknown, approximate forms are required in practice, and the accuracy of results is limited by the quality of these approximations.

The development of accurate XC functionals has been an important subject for decades (*8*–*10*). To date, researchers have proposed more than 200 different XC functionals (*11*). Most functionals used today contain a few to a few dozens of empirical parameters, which are usually determined by fitting to datasets of molecular or materials properties. Many widely used XC functionals, such as those developed by Head-Gordon and co-workers (*12*–*16*) and the well-known Minnesota functionals (*17*–*20*), are constructed by taking linear combinations of expressions inspired by existing functional forms [e.g., the B97 functional (*21*)], where the linear coefficients and other empirical parameters are fit to databases such as the Main Group Chemistry Database (MGCDB84) (*15*) and Minnesota Database (*22*).

Despite great efforts, it is generally considered difficult to develop more accurate functionals than existing ones in a systematic manner. In the past decade, researchers have devoted great efforts to approximate functionals using machine learning (ML) (*23*). One direction is to accelerate DFT with accurate kinetic energy functional approximation or bypass the KS equations using kernel ridge regression (*24*–*28*) and neural networks (*29*–*32*). The other direction is to solve the decades-long challenge—fundamentally improving the accuracy of DFT with better XC functionals. Various ML techniques have been applied, e.g., Bayesian error estimation (*33*, *34*), linear regression with subset selection procedure (*13*–*16*), genetic algorithm (*35*), and Bayesian optimization (*36*). In these works, the functional forms are usually chosen a priori or selected from a relatively rigid space of functional forms. Furthermore, many parameters in these forms are linear in nature, which has the advantage of being easily optimizable but limits the expressive power of the functional form. In contrast, neural networks are able to approximate any continuous function (*37*) and thus are flexible approximators to parameterize XC functionals. These neural networks can be trained with self-consistent DFT calculations via differentiable programming (*38*–*40*), via simulated annealing (*41*, *42*), or on converged DFT or beyond DFT calculations (*43*–*45*). Although neural-network XC functionals with more than tens of thousands of parameters can achieve high accuracy for particular systems, they are less explainable to humans, expensive to execute, and difficult to integrate to existing DFT codes compared to conventional human-designed symbolic forms.

**Fig. 1.**
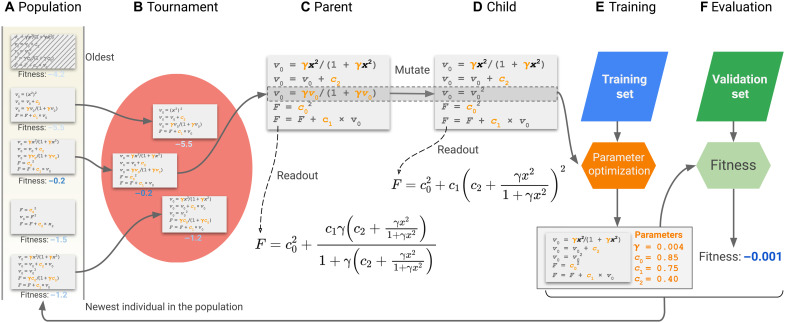
Workflow of the SyFES framework. (**A**) A population of symbolic density functionals is iteratively evolved using the regularized evolution algorithm. Each individual in the population represents the enhancement factors in a density functional. The performance of a density functional is characterized by its fitness, defined as the negative of validation error in kilocalories per mole. (**B**) In each iteration, a tournament selection is performed on a subset of individuals sampled from the population. (**C**) The highest-fitness individual in the tournament is selected to be the parent. (**D**) Then, a child functional is generated by randomly picking one of the enhancement factors and mutating one of its instructions. (**E**) The child functional is then trained on the training set using the covariance matrix adaptation evolution strategy (CMA-ES) method to determine its parameters. (**F**) Last, the fitness of child functional is evaluated on the validation set and then added to the population. After the population size exceeds a limit, the oldest individual in the population is removed. For simplicity of visualization, a density functional is represented with only one enhancement factor. The general form of density functional considered in this work contains three enhancement factors (*F*_x_, *F*_c−ss_, and *F*_c−os_), and in each evolution step, one enhancement factor is randomly chosen for mutation.

Here, we propose a new approach to develop more accurate functionals—searching XC functionals in a large, nonlinear, symbolic functional space based on the concept of symbolic regression. Unlike most ML methods where models are formulated numerically, symbolic regression outputs the resulting model in the symbolic form. Recently, there is emerging interest in the development of symbolic regression methods for physical science problems (*46*–*56*). We illustrate our framework in Fig. 1 and denote it as Symbolic Functional Evolutionary Search (SyFES). One key component of SyFES is a symbolic representation of XC functionals based on elementary mathematical instructions and building blocks of existing functionals. The symbolic representation mimics the execution of XC functionals by computer programs, and we demonstrate that this representation enables efficient search of functional forms. Then, using a genetic algorithm called regularized evolution (*57*), we demonstrate that simple functionals such as the B97 exchange functional can be obtained from scratch and that more accurate functionals can be obtained by evolving from existing functionals. In particular, from a set of regularized evolution starting from the ωB97M-V functional (*15*), we found a functional form, GAS22 (Google Accelerated Science 22), with lower test error on the MGCDB84 dataset, which is the dataset that the ωB97M-V functional is originally trained on. We further demonstrate that GAS22 exhibits good numerical stability for self-consistent calculations.

## RESULTS

### Representation of XC functionals

In KS-DFT, the total energy *E*_tot_ for a system of interacting electrons is represented as a functional of electron density ρ

$$E_{\text{tot}}[\rho ]=T_{s}[\rho ]+E_{\text{ext}}[\rho ]+E_{H}[\rho ]+E_{\text{xc}}[\rho ]$$ (1)

where *T*_s_[ρ], *E*_ext_[ρ], *E*_H_[ρ], and *E*_xc_[ρ] denote the KS kinetic energy, the external energy, the Hartree energy, and the XC energy, respectively. Only the exact form for *E*_xc_[ρ] is unknown, and approximate forms must be used in practice. Most existing approximate forms for *E*_xc_[ρ] contain a semilocal part $E_{\text{xc}}^{\text{sl}}$, and many modern functional forms also contain a nonlocal part $E_{\text{xc}}^{\text{nl}}$. The overall *E*_xc_ can thus be written as

$$E_{\text{xc}}[\rho (r)]=E_{\text{xc}}^{\text{sl}}[\rho (r)]+E_{\text{xc}}^{\text{nl}}[\rho (r)]$$ (2)

The semilocal part $E_{\text{xc}}^{\text{sl}}$ can generally be written as an integral of XC energy density *e*_xc_ over real-space coordinate ***r***, with *e*_xc_ being a function of ρ and its various orders of derivatives. The semilocal part is generally the energetically dominant component of *E*_xc_ and is the main focus of this work. The nonlocal part $E_{\text{xc}}^{\text{nl}}$ is usually introduced to address certain interactions that are difficult to capture by the semilocal part, e.g., dispersion interaction.

We fix the nonlocal part $E_{\text{xc}}^{\text{nl}}$ being identical to that of the ωB97M-V functional and search for better semilocal part $E_{\text{xc}}^{\text{sl}}$. For simplicity, we present formulas for spin-unpolarized systems in this section, and in the Supplementary Materials, we present the general formalism for spin-polarized systems. In the semilocal part

$$E_{\text{xc}}^{\text{sl}}[\rho ]=\int (e_{x-\text{sr}}^{\text{LDA}}[\rho ]F_{x}+e_{c-\text{ss}}^{\text{LDA}}[\rho ]F_{c-\text{ss}}+e_{c-\text{os}}^{\text{LDA}}[\rho ]F_{c-\text{os}})dr$$ (3)

terms $e_{x-\text{sr}}^{\text{LDA}}[\rho ]$, $e_{c-\text{ss}}^{\text{LDA}}[\rho ]$, and $e_{c-\text{os}}^{\text{LDA}}[\rho ]$ denote short-range exchange, same-spin correlation, and opposite-spin correlation energy densities within the local density approximation (LDA) (*58*), respectively. Their explicit forms are known [see the Supplementary Materials for the expression of $e_{x-\text{sr}}^{\text{LDA}}$ and (*59*) for expressions of $e_{c-\text{ss}}^{\text{LDA}}$ and $e_{c-\text{os}}^{\text{LDA}}$]. *F*_x_, *F*_c−ss_, and *F*_c−os_ are the exchange, same-spin correlation, and opposite-spin enhancement factors that represent corrections over LDA energy densities. The enhancement factors can depend on density gradient for generalized gradient approximation (GGA) functionals and additionally on kinetic energy density for meta-GGA functionals. This form has been adopted by many functionals, most notably the B97 functional (*21*) and many B97-inspired functionals (*12*–*20*). The functional form in Eq. 2 is then entirely determined by enhancement factors (*F*_x_, *F*_c−ss_, and *F*_c−os_). Thus, we will use the term XC functional and enhancement factors interchangeably in the following discussions. We note that, although we selected a particular form of $E_{\text{xc}}^{\text{sl}}$ and $E_{\text{xc}}^{\text{nl}}$, the SyFES framework is general and can be applied to other forms.

### Symbolic representation for machine

Human representation of the XC functionals is symbolic. For machines to automatically search the symbolic forms of the XC functionals, we need a machine representation that can both be translated to the human representation and be easily modified by search algorithms. In this work, we represent the form of XC functionals as the execution of a set of mathematical instructions. This representation is inspired by recent progress in the field of automated ML (AutoML) (*60*, *61*). For instance, Real *et al*. (*60*) showed that, by representing a computer program as a sequence of instructions, a regularized evolution algorithm (*57*) can learn to construct complicated computer programs from a set of instructions for various ML tasks. Notably, it can automatically rediscover many ML techniques that researchers developed and used in the past decades.

To represent XC functionals using instructions, we define a workspace containing features, variables, and parameters. The features represent the input of the functional form (enhancement factors) such as electron density, density gradient, etc. The variables store intermediate variables during functional execution and are all initialized to zero. *F* is a special variable in the workspace, whose value is taken as the resulting enhancement factor after all the instructions are executed. The parameters represent scalars that should be optimized by fitting to the training dataset after the symbolic form is determined. Then, these parameters are considered constants in the functional evaluations. This definition of workspace mimics the memory space when using a computer program to evaluate the density functional.

**Fig. 2.**
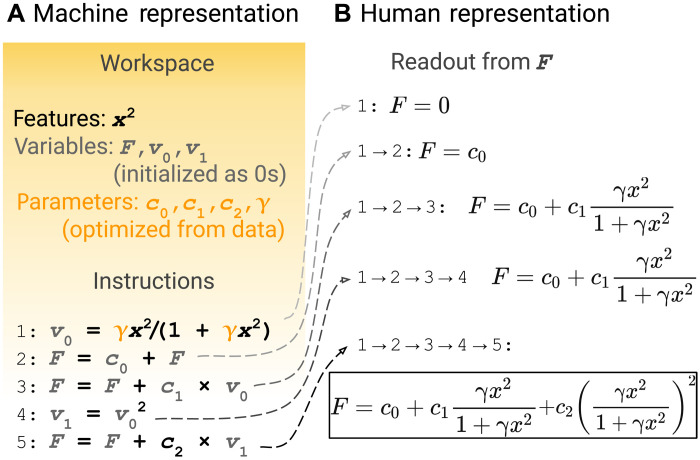
Representation of symbolic functionals. (**A**) Machine representation of the symbolic functionals. The B97 enhancement factor is represented as five consecutive instructions operating on a workspace. (**B**) The intermediate readout from *F* during the consecutive execution of the instructions. The final readout (boxed) is the enhancement factor.

We use the B97 functional as an example to illustrate this representation. In the B97 functional, enhancement factors *F*_x_, *F*_c−ss_, and *F*_c−os_ take the form *F*^B97^ = *c*_0_ + *c*_1_*u* + *c*_2_*u*^2^, where the auxiliary quantity *u* = γ*x*^2^/(1 + γ*x*^2^) is a finite-domain transform of the reduced density gradient *x* = 2^1/3^∣∇ρ∣/ρ^4/3^. In Fig. 2A, the B97 enhancement factor is written as five consecutive mathematical instructions operating on a workspace composed of one feature (the square of the reduced density gradient, *x*^2^), four parameters (*c*_0_, *c*_1_, *c*_2_, and γ), and three variables (*v*_0_, *v*_1_, and *F*). Then, we execute the instructions consecutively and then read out the final value from *F*. Figure 2B lists the intermediate readout from *F* to explain how *F* changes during the execution. After the execution of all the instructions, the readout from the variable *F* equals to *F*^B97^ = *c*_0_ + *c*_1_*u* + *c*_2_*c*^2^, where $u=\frac{\gamma x^{2}}{1+\gamma x^{2}}$. Here, we consider three types of instructions: arithmetic operations (e.g., addition), power operations (e.g., square), and building blocks from existing functional forms (e.g., the γ[·]/(1 + γ[·]) operation in the B97 form); see Table 1 for details. We remark that, throughout this work, we do not presume any parameters to be linear (e.g., parameters *c*_0_, *c*_1_, and *c*_2_ in *F*^B97^), and we generally treat all parameters as nonlinear parameters during the parameter optimization process.

**Table 1.** Instructions for symbolic functional representation. We consider arithmetic operations, powers, and building blocks from existing functionals. *p*, *q*, and *s* denote symbols in the workspace, where the right-hand side symbols (*p* and *q*) can be any features, parameters, or variables, and the left-hand side symbol (*s*) can be any variables. A subset of four instructions [*s* = *p* + *q*, *s* = *s* + *p* × *q*, *s* = *p*^2^, and *s* = γ*p*/(1 + γ*p*)] is used in proof-of-principles calculations for the B97 exchange functional.

**Arithmetic operations**	
*s = p + q*	*s = p − q*
*s* = *p* × *q*	*s* = *p*/*q*
*s* = *s* + *p*× q^*^	
**Power operations**	
*s* = *p^n^*, *n* ∈ {2,3,4,6,1/2,1/3}	
**Building blocks from existing functionals^†^**	
*s* = γ*p*/(1 + γ*p*), γ is a parameter	

*This instruction denotes the *s* + = *p* × *q* instruction, where += is the addition-assignment operator in common programming languages. The inclusion of this instruction can reduce the number of instructions required to represent functionals such as B97 or ωB97M-V.

†This instruction comes from the B97 functional. Several other building blocks are also explored but are found to play insignificant roles; see the Supplementary Materials for details.

The workspace and instructions define the search space of the symbolic functionals. Then, we chose a set of four mutation rules to modify a symbolic functional: the insertion of a new instruction, the removal of an existing instruction, the change of operation in an instruction, and the change of argument in an instruction. These mutation rules enable the evolutionary search procedure to generate new symbolic forms starting from existing ones, thus exploring the search space of functional forms.

### Symbolic Functional Evolutionary Search

After the symbolic representation of functionals is determined, the problem of searching functionals is transformed into a combinatorial optimization of instructions, features, variables, and parameters. We designed a framework, SyFES, to construct the symbolic functional forms that best fit the data (Fig. 1). In particular, the regularized evolution algorithm (*57*) maintains and evolves a population of individuals based on the fitness of individuals. In each iteration, a random subset of individuals is drawn from the entire population, and the individual with the highest fitness is chosen (tournament selection) as the parent, which is then mutated to generate a child and added back to the population. When the size of the population grows beyond a limit, the oldest individual is removed from the population. Compared to standard genetic algorithms, which usually remove the individual with the lowest fitness, the regularized evolution algorithm introduces aging to avoid individuals with good fitness but poor robustness to stay in the population for an excessive amount of time. It leads to better exploration of the search space and better generalization of the best individuals it found.

In this work, a symbolic functional form is treated as an individual. The initial population can be the empty functional or existing functionals. Each time a new functional is obtained, its scalar parameters are determined by minimizing the training error *J*_train_. We adopt the weighted root mean square deviation (WRMSD) from (*15*) as the objective function

$$J_{S}=\sqrt{\frac{1}{N}\sum_{i\in S}w_{i}{\left(E_{i}-E_{i}^{\text{ref}}\right)}^{2}}$$ (4)

where *N* is the total number of data points in the dataset *S* (training, validation, or test). *E_i_* and $E_{i}^{\text{ref}}$ are the *i*th energetic data and its reference value, respectively. *E_i_* can generally be computed from the DFT total energies (*E*_tot_ in Eq. 1) of one or more molecules. For instance, an *E_i_* that corresponds to isomerization energy is computed by taking the difference of *E*_tot_ between two isomers. *w_i_* is the sample weight that accounts for the different scales of energetic data in different subsets.

After the scalar parameters are determined, SyFES records the fitness of the new functional as −*J*_val_, which is used later in the tournament selection. As the procedure advances, the population gradually evolves to functionals with higher fitness (lower validation error). The functional form with the highest fitness is selected as the final output from SyFES.

In the following two subsections, we present two demonstrations of the SyFES framework. In the first proof-of-principle demonstration, SyFES is capable of finding a known functional (B97 exchange functional) from scratch. In the second demonstration, SyFES can evolve from an existing functional (ωB97M-V) to a novel functional form GAS22, which has better performance (test error) on the MGCDB84 dataset. In both demonstrations, we use the electron densities evaluated from the ωB97M-V functional as input to Eq. 1 to evaluate each new functional form to accelerate the search. At the end, we present self-consistent DFT calculations using GAS22.

### Rediscovery of B97 exchange functional

As a proof-of-principle demonstration, we apply SyFES to a case where the ground-truth functional is known. In particular, we adopt the TCE [thermochemistry (easy)] subset of MGCDB84 that contains 947 data points for thermodynamics of molecules, and we randomly partition the 947 data points into training, validation, and test set, which contain 568, 189, and 190 data points, respectively. We set the reference energies in Eq. 4 to the total energies evaluated using the B97 exchange functional. In this demonstration, we verify whether SyFES is capable of finding the B97 enhancement factor *F*^B97^ or its equivalence.

We design the search space to be functionals that can be represented by less or equal to six instructions from a set of four instructions *s* = *p* + *q*, *s* = *s* + *p* × *q*, *s* = *p*^2^, and *s* = γ*p*/(1 + γ*p*). The workspace contains one feature (*x*^2^), four parameters, and three variables. The number of parameters is chosen corresponding to the parameters *c*_0_, *c*_1_, *c*_2_, and γ, as shown in Fig. 2A. Except for γ, which is bound to the finite-domain transform, all other parameters, together with features and variables, are free to use as arguments in any instructions.

**Fig. 3.**
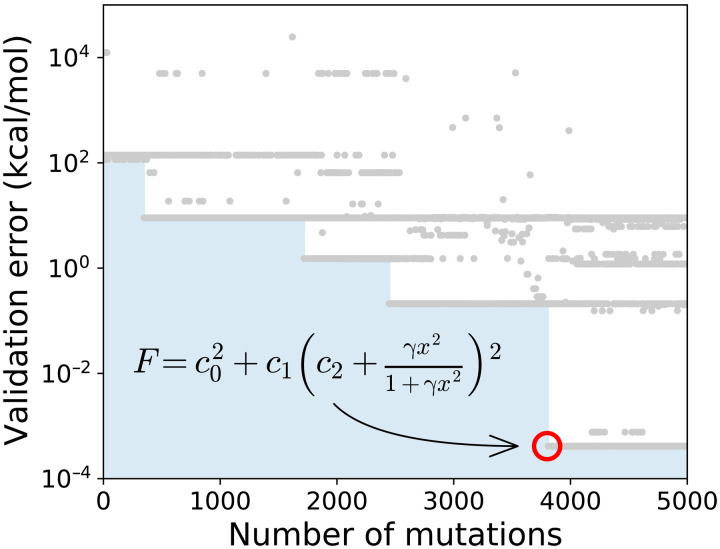
Validation error of symbolic functionals generated by SyFES starting from an empty functional (no instruction). The total energies evaluated using the B97 exchange functional are used as reference energies for training and evaluation of functionals. Each gray dot represents one functional form generated by SyFES. The blue area represents the cumulative minimum validation error up to a certain number of mutations. After less than 4000 mutations, an equivalent form of B97 enhancement factor (red circle) is obtained.

We ran SyFES starting from scratch, where the initial functional contains no instructions and thus constantly outputs zero. Figure 3 shows the validation error as a function of the number of mutations, where each dot corresponds to a functional form. After less than 4000 iterations, SyFES was able to find a functional form with validation error *J*_val_ = 4.2 × 10^−4^ kcal/mol and test error *J*_test_ = 3.7 × 10^−4^ kcal/mol. The functional has the following symbolic form

$$F=c_{0}^{2}+c_{1}{\left(c_{2}+\frac{\gamma x^{2}}{1+\gamma x^{2}}\right)}^{2}$$ (5)

with *c*_0_ = 0.8504, *c*_1_ = 0.7480, *c*_2_ = 0.3394, and γ = 0.0040. It is easy to verify that this functional form is equivalent to the B97 one: $F_{x}^{B97}=c_{0}+c_{1}\frac{\gamma x^{2}}{1+\gamma x^{2}}+c_{2}{\left(\frac{\gamma x^{2}}{1+\gamma x^{2}}\right)}^{2}$ with *c*_0_ = 0.8094, *c*_1_ = 0.5073, *c*_2_ = 0.7481 and, γ = 0.004. This study demonstrates that SyFES can find existing simple symbolic functional forms from scratch.

### Evolving from the ωB97M-V functional

Now, we turn to the main result of this work, where we demonstrate that SyFES is capable of evolving from existing functional forms to novel functional forms. We use the ωB97M-V functional as a starting point. Its enhancement factors can be written as power series in two variables

$$F^{\omega B97M-V}=\sum_{i}\sum_{i}c_{\mathrm{ij}}w^{i}u^{j}$$ (6)

where *w* is an auxiliary quantity related to the kinetic energy density τ and *u* is the auxiliary quantity related to reduced density gradient *x* as in the case of the B97 functional (see the Supplementary Materials for detailed definitions). *c_ij_* are linear coefficients for the power series of *w* and *u*. In the ωB97M-V functional, the exchange enhancement factor $F_{x}^{\omega B97M-V}$ includes *c*_00_, *c*_10_, and *c*_01_ terms; the same-spin correlation enhancement factor $F_{c-\text{ss}}^{\omega B97M-V}$ includes *c*_00_, *c*_10_, *c*_20_, *c*_43_, and *c*_04_ terms; and the opposite-spin correlation enhancement factor $F_{c-\text{os}}^{\omega B97M-V}$ includes *c*_00_, *c*_10_, *c*_20_, *c*_60_, *c*_21_, and *c*_61_ terms. These terms were determined through a best subset selection procedure using the training and validation set of MGCDB84 (*15*). Overall, there are 15 linear parameters (*c_ij_*’s) and three nonlinear parameters (the γ parameters in the definition of *u* for *F*_x_, *F*_c−ss_, and *F*_c−os_) in the semilocal part of the ωB97M-V functional. The linear parameters are determined by performing linear regression on the MGCDB84 training set, and the nonlinear parameters are directly taken from previous studies.

To use the ωB97M-V functional as the starting point in SyFES, we first represent it as consecutive instructions. We adopted all instructions shown in Table 1, which leads to a substantially larger search space than the proof-of-principles demonstration on the B97 exchange functional. In addition to instructions used for searching the B97 exchange functional, we also include subtraction, division, and powers with additional exponents. Besides arithmetic operations, we also explored using existing functional building blocks from existing functionals, but we found that those forms are too restrictive to be selected by the SyFES. Using such a choice of instruction set, $F_{x}^{\omega B97M-V}$, $F_{c-\text{ss}}^{\omega B97M-V}$, and $F_{c-\text{os}}^{\omega B97M-V}$ can be represented using 6, 11, and 11 instructions, respectively (see the Supplementary Materials for the symbolic representation of ωB97M-V).

**Fig. 4.**
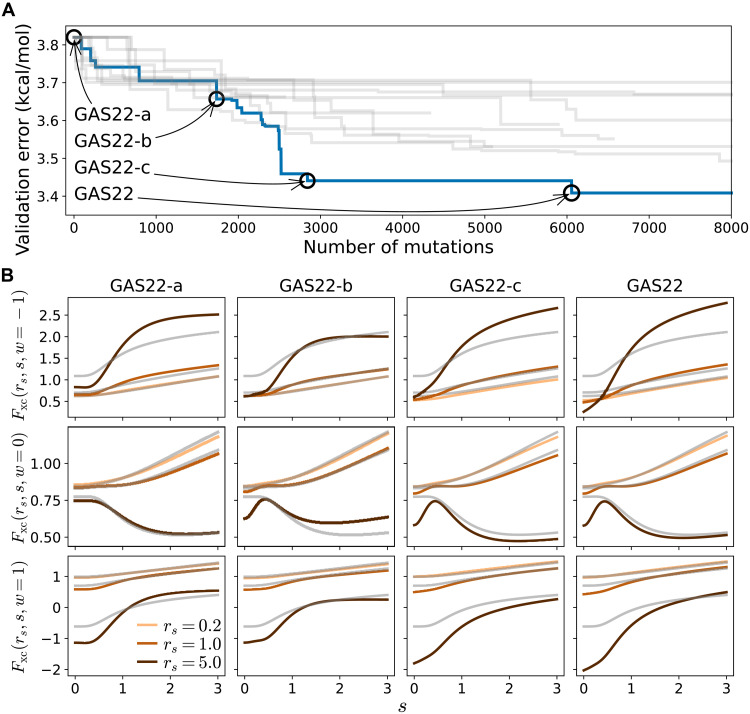
Evolving functional forms starting from ωB97M-V. (**A**) Validation error of symbolic functionals generated by SyFES. Different curves denote different independent evolutions; the evolution trajectory with the optimal–validation error individual is colored blue. (**B**) XC enhancement factors *F*_xc_ (see text) of four snapshots of functionals in the best evolution. *F*_xc_ is plotted as functions of dimensionless quantities *s* = *x*/2(3π^2^)^1/3^ and *w* at different Wigner-Seitz radius *r_s_* = (3/4πρ)^1/3^. Gray curves denote the enhancement factors of ωB97M-V functional.

On the basis of this representation, we performed a set of 12 independent evolutions starting from the ωB97M-V functional. These evolutions explored 29,628 functional forms in total. Each curve in Fig. 4A presents the cumulative minimum validation error of symbolic functionals explored in one evolution. Once a new functional form is obtained with lower validation error than all the previous functionals, the plot has a step down. The evolution leading to the functional with the lowest validation error is marked in blue. The figure shows that SyFES is able to generate new functional forms with decreasing validation error as the evolution proceeds. For comparison, we also performed a set of random search studies that randomly mutate functionals from the population without the tournament selection. The random search cannot effectively obtain functionals with improved performance (more details in the Supplementary Materials).

Now, we turn to the behavior of functionals. We denote the best functional obtained by the end of the evolution as GAS22. In addition, we pick three precedent functional forms GAS22-a, GAS22-b, and GAS22-c along the best trajectory in Fig. 4A. To illustrate their numerical behavior, in Fig. 4B, we present their XC enhancement factor $F_{\text{xc}}=e_{\text{xc}}/e_{\text{xc}}^{\text{LDA}}=(e_{x}^{\text{LDA}}F_{x}+e_{c-\text{ss}}^{\text{LDA}}F_{c-\text{ss}}+e_{c-\text{os}}^{\text{LDA}}F_{c-\text{os}})/(e_{x}^{\text{LDA}}+e_{c-\text{ss}}^{\text{LDA}}+e_{c-\text{os}}^{\text{LDA}})$, which characterize the deviation of XC energy density from that of the LDA. Because we are training a meta-GGA functional, *F*_xc_ would depend on density, density gradient, and kinetic energy density. The *F*_xc_ of ωB97M-V is plotted in gray for comparison. The first, second, and third rows show the behavior of *F*_xc_ at *w* = {− 1,0,1}, which correspond to chemical bonds with weak, metallic, and covalent characters, respectively (*62*). In each subplot, we plot *F*_xc_ as a function of *s* = *x*/2(3π^2^)^1/3^ at multiple values of Wigner-Seitz radius *r_s_*. *s* is proportional to the reduced density gradient *x* and is a common auxiliary quantity used in literature for analyzing density functionals. Normal physical systems usually have *s* between 0 and 3 (*63*). The Wigner-Seitz radius *r_s_* = (3/4πρ)^1/3^ characterizes the electron density, where a larger value of *r_s_* corresponds to lower electron density. On the basis of the plots, one can see that the GAS22 (brown curves) differ from ωB97M-V (gray curves) in a few regions. The first regime involves small density gradient (*s* < 1), where *F*_xc_ of GAS22 tends to be lower than ωB97M-V. The second regime involves weak bonds (*w* = −1) and small electron density (*r_s_* = 5), where *F*_xc_ of GAS22 tends to be higher than ωB97M-V.

After simplification, the final symbolic form of GAS22 is

$$F_{x}=0.862+0.937u+0.318w$$ (7)

$$F_{c-\text{ss}}=u-4.108w-5.242w^{2}-1.766u^{6}+7.538w^{4}u^{6}$$ (8)

$$F_{c-\text{os}}=0.805+7.989w^{2}-7.548w^{6}+2.001w^{6}\sqrt[{3}]{x^{2}}-1.761w^{2}\sqrt[{3}]{x^{2}}$$ (9)

where *u* = γ*x*^2^/(1 + γ*x*^2^), similar to the B97 and the ωB97M-V functional, with γ = 0.00384 in *F*_x_ and γ = 0.469 in *F*_c−ss_. The exchange enhancement factor in Eq. 7 is symbolically identical and numerically similar (fig. S1, last column) to the ωB97M-V functional, which indicates that the ωB97M-V exchange enhancement factor $F_{x}^{\omega B97M-V}$ may be accurate enough for depicting the exchange. The best subset selection presented in (*15*) only selected the three lowest-order terms for the definition of $F_{x}^{\omega B97M-V}$, indicating that the exchange functional is easily captured by the form of two-dimensional power series in *u* and *w*. SyFES recognized this and maintained the symbolic form of the exchange functional. The same-spin correlation enhancement factor in Eq. 8 still assumes the form of power series in two variables, but the orders are no longer those in ωB97M-V, indicating that the symbolic regression is capable of applying minor symbolic modifications to existing forms for lower error. The most notable difference is found in the opposite-spin correlation enhancement factor in Eq. 9, which contains a novel *x*^3/2^ term that is completely outside of the space spanned by power series in *u* and *w* as in Eq. 6. It highlights the power of SyFES in the discovery of novel functional forms from data. To assess the performance of the functional, we apply it to the test set of MGCDB84, which was not used during the training and validation of functional forms. The test error of GAS22 is 3.585 kcal/mol, a 15% improvement over the ωB97M-V functional (4.237 kcal/mol).

### Self-consistent calculations using GAS22

**Fig. 5.**
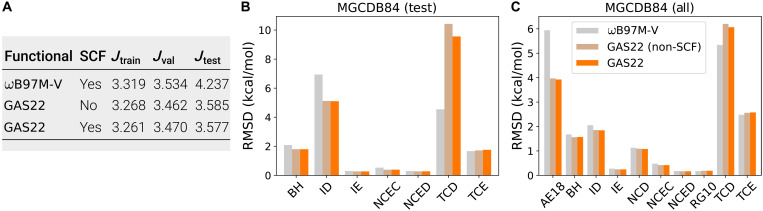
Performance of the functionals on the MGCDB84 dataset. (**A**) Training, validation, and test WRMSD (kcal/mol) of GAS22 functional on the MGCDB84 dataset. Non-SCF results are evaluated using ωB97M-V density. (**B**) RMSD on the test set of MGCDB84 dataset. (**C**) RMSD on the entire MGCDB84 dataset. AE18, atomic energy; BH, barrier height; ID, isomerization energies (difficult); IE, isomerization energies (easy); NCEC, noncovalent clusters (easy); NCED, noncovalent dimers (easy); NCD, noncovalent dimers (difficult); RG10, rare gas dimers.

So far, all the results presented are based on non–self-consistent calculations on ωB97M-V densities. To evaluate the performance of GAS22 in realistic DFT calculations, we performed self-consistent field (SCF) calculations where the functional derivatives are computed using automatic differentiation. Figure 5A presents the training, validation, and test errors of GAS22 after performing SCF calculations. SCF results are very similar to non-SCF ones, demonstrating good numerical stability of GAS22 found by SyFES.

The training, validation, and test error were computed as WRMSD defined in Eq. 4, with weights reported in (*15*) (see Materials and Methods for additional details). Because the same weights were used to compute the training and validation errors for the development of ωB97M-V functional, we use WRMSD on the test set as a general summary of the overall performance. We understand the insufficiency of using a scalar to measure the performance of functionals on diverse subsets. Therefore, to further benchmark the performance of GAS22 on different types of molecules, in Fig. 5 (B and C), we report the RMSD of the GAS22 and the ωB97M-V functional on subsets of MGCDB84. We see that GAS22 outperforms ωB97M-V for most subsets. The only subset where GAS22 shows a less favorable RMSD than ωB97M-V is the TCD [thermochemistry (difficult)] subset, which is composed of strongly correlated molecules. The comparison between the SCF results of GAS22 and ωB97M-V demonstrates that, despite not using SCF calculations during the evolution, SyFES is capable of finding functional forms with good performance in realistic SCF calculations.

## DISCUSSION

We proposed a ML approach for developing accurate XC functionals, in contrast to conventional human-designed symbolic functionals and other ML-generated numerical functionals. SyFES can automatically search functionals that best fit the given dataset from a large, nonlinear, symbolic functional space. As demonstrated, it is capable of finding simple existing functionals from scratch, as well as evolving an existing functional to a better performing functional. Despite the fact that the search procedure is conducted by computers, it is worth noting that the form of functionals produced by SyFES has similar simplicity as functionals designed by humans in the past few decades—symbolic forms with a manageable amount of parameters. Thus, they are amenable to all the interpretation methods scientists developed to examine the properties of functionals and understand their limitations. Meanwhile, the computational cost to use and the work to implement these functionals in popular libraries such as Libxc (*64*) are also the same as the conventional functionals on the same level of Jacob’s ladder (*65*). SyFES is ML applied to developing human-readable scientific expressions and not just a blackbox prediction.

Given the ubiquity of DFT in quantum simulations, we expect many applications along this direction in chemistry, physics, and materials sciences. This work focused on main-group chemistry using the data from the MGCDB84 dataset. However, the framework itself is general and can be applied to more systems via incorporating a new dataset, objective function, and search space. The design of SyFES is highly scalable on computer clusters and cloud platforms, where the mutation, training, and validation of new functionals are distributed into different workers asynchronously. In this work, we used up to 50 workers, but it can easily be scaled up to leverage more computing power to deal with a larger search space for more demanding problems. We conclude with a list of promising future workstreams: (i) Better dataset. It has been proved many times that high-quality and comprehensive datasets are critical to the development and benchmarking of ML algorithms for target applications. For example, ImageNet (*66*) spawned the revolution of ML in computer vision. This area is underinvested for the ML DFT community. (ii) Refined methodology. Starting from the results here, SyFES can be further improved in multiple dimensions, e.g., search space design, feature engineering, regularization via numerical techniques, symbolic constraints, or adding density information. (iii) Richer applications in density functionals. It includes developing highly accurate empirical XC functionals for a family of systems, pursuing the universal functional with the attempts to include more exact conditions, constructing accurate kinetic energy functionals for large-scale systems in orbital-free DFT, and formulating classical DFT (*67*, *68*) for fluids or even crowds (*69*).

Could SyFES or its successors replace human in the development of functionals? Conversely, humans have a long history of using computers to assist scientific discovery and recent advances in ML-guided mathematicians finding new insights in topology andrepresentation theory (*70*). SyFES may help scientists focus more on the physics insights and quality of functionals. This research direction paves the way of efficient development of density functionals using advanced algorithms and computing power.

## MATERIALS AND METHODS

### Dataset and objective function

In this work, we use the Main Group Chemistry Database (MGCDB84) to train and evaluate functional forms. The training, validation, and test set used in this work correspond to the training, primary test, and secondary test set in (*15*). The objective function defined in Eq. 4 is identical to (*15*), thus facilitating a direct comparison between the symbolic functionals obtained in this work and ωB97M-V.

The MGCDB84 dataset contains nine types of molecular energetics data: AE18 (atomic energies), NCED [noncovalent dimers (easy)], NCEC [noncovalent clusters (easy)], NCD [noncovalent dimers (difficult)], IE [isomerization energy (easy)], ID [isomerization energy (difficult)], RG10 (rare gas potential energy curves), TCE, TCD, and BH (barrier heights), where the difficult/easy in the names represents the presence/lack of interactions that are difficult to capture by KS-DFT, such as strong electron correlation. There are 4986 data points in total in the dataset, with accuracy estimated to be at least 10 times more accurate than the best available DFT calculations. Most data points in the dataset are relative energy differences between different molecular species, and 5931 single-point DFT calculations are required to evaluate the 4986 data points. The proof-of-principle calculations targeting B97 exchange functional use the TCE subset (947 data points) and a standard 60-20-20% splitting for constructing training, validation, and test sets.

For evolution studies starting from ωB97M-V, we used the same training, validation, and test sets as (*11*, *15*), which contains 870, 2960, and 1150 data points, respectively. We note that this grouping is different from standard ML practice, which assumes that the training, validation, and test sets are drawn from the same distribution. In this partition, the training, validation, and test set each include different subsets of molecular properties, which poses stronger criteria for the transferability of functionals trained on the dataset. For the calculation of training, validation, and test error as defined in Eq. 4, we used the following weights (*15*) for different data types: 0.1 for TCD; 1 for TCE and AE18; 10 for NCD, ID, and BH; 100 for NCE and IE; and 10,000 for RG10.

### Regularized evolution

Regularized evolution is performed with a massively parallel implementation (see the Supplementary Materials for the software design). The implementation takes advantage of just-in-time compilation (*71*) to enable training of functional forms on graphic processing units (GPUs). The population (Fig. 1A) of functional forms is managed in a central processing unit (CPU) server. Each evolution uses 50 parallel GPU workers to evolve functionals (Fig. 1, B to F) asynchronously. All evolutions adopt a maximum population size of 100 and a tournament selection size of 25, except for the evolution that targets the B97 exchange functional, which uses a tournament size of 10.

In the symbolic representation used in this work, one functional form may have multiple equivalent symbolic representations. For example, a mutation may introduce instructions that have no effect in the current functional, although these instructions may become useful in subsequent mutations. We designed a functional equivalence checking mechanism to circumvent the duplicated training and validation of equivalent functional forms (see the Supplementary Materials for details), which accelerate the functional search by an order of magnitude.

### Parameter optimization

After mutation, the scalar parameters in the new functional form are optimized to minimize the training error *J*_train_. The optimization is performed using the covariance matrix adaptation evolution strategy (CMA-ES) method (*72*). A CMA-ES optimization proceeds by iteratively optimizing a population of parameters, with the covariance matrix characterizing the spread of the population updated on the fly during the optimization process. In general, for a given functional form, there may be different choices of parameters that all lead to low training error (i.e., multiple local minima). For each functional form, the CMA-ES optimization is performed multiple times with initial guess for parameters randomly drawn from a unit Gaussian distribution, and the parameters leading to the lowest training error are adopted. The optimization for each functional is repeated 10 times in the evolution for B97 exchange functional and 5 times for evolutions starting from ωB97M-V functional. Because of the flexible nature of functional forms generated in this work, we constrained the value of all the parameters to [−10, 10] to avoid overfitting and numerical instability.

### Self-consistent DFT calculations

Self-consistent DFT calculations are performed by solving the KS equations

$$-\nabla^{2}/2+v_{\text{ext}}+v_{H}+v_{\text{xc}}\psi_{i}(r)=\varepsilon_{i}\psi_{i}(r)$$ (10)

where ψ*_i_* denotes the KS orbitals. *v*_ext_, *v*_H_, and *v*_xc_ denote the external, Hartree, and XC potential, respectively. The XC potential *v*_xc_ is defined as the functional derivative of the XC functional *E*_xc_ with respect to the electron density ρ(***r***)

$$v_{\text{xc}}(r)=\frac{\delta E_{\text{xc}}[\rho (r)]}{\delta \rho (r)}$$ (11)

where the density $\rho (r)=\sum_{i}^{\text{occ}}{|\psi_{i}(r)|}^{2}$ sums overoccupied KS orbitals. For self-consistent calculations with the GAS22 functional, the functional derivative in Eq. 11 is evaluated through automatic differentiation using the JAX package (*71*).

All self-consistent DFT calculations are performed with the PySCF package (*73*) with a large basis set def2-QZVPPD (*74*). The default integration grid in PySCF is adopted for the evaluation of semilocal XC energies; SG-1 prune (*75*) is used for evaluating VV10 (*76*) nonlocal correlation energies.

Of the 5931 single-point DFT calculations needed to evaluate the entire MGCDB84 dataset, there are 7/8 single-point SCF calculations with the ωB97M-V/GAS22 functional that did not achieve convergence, which affects the evaluation of the 6/7 data points of all the 4986 data points. Using reference values for ωB97M-V results reported in (*15*), we estimate that excluding these data points leads to less than 0.01% change in the calculation of training, validation, and test errors.

## Supplementary Materials

### This PDF file includes:

Sections S1 to S7 Figs. S1 to S5 References
