# Supplementary material for: Evolving symbolic density functionals
Source: Sci Adv. 2022 Sep 9;8(36):eabq0279. doi: 10.1126/sciadv.abq0279 (PMC9462698; doi:10.1126/sciadv.abq0279)
Supplement: Supplementary file 1 — Sections S1 to S7 Figs. S1 to S5 References [file sciadv.abq0279_sm.pdf]

Science Advances
AAAS

Supplementary Materials for

Evolving symbolic density functionals

He Ma *et al.*

Corresponding author: Email: Li Li, leeley.lili@gmail.com

Sci. Adv. **8**, eabq0279 (2022)
DOI: 10.1126/sciadv.abq0279

This PDF file includes:

Sections S1 to S7
Figs. S1 to S5
References

Supplementary Materials

1 Functional forms

In this section we present the functional forms in main text Eq. 2-3 for general systems (which may contains spin polarization).

The semilocal part of the exchange-correlation functional assumes the following form

$$E_{\mathrm{xc}}^{\mathrm{sl}} = \int \left(\sum_{\sigma} e_{\text{x-sr},\sigma}^{\mathrm{LDA}} F_{\mathrm{x},\sigma} + \sum_{\sigma} e_{\text{c-ss},\sigma}^{\mathrm{LDA}} F_{\text{c-ss},\sigma} + e_{\text{c-os}}^{\mathrm{LDA}} F_{\text{c-os}} \right) \mathrm{d}\boldsymbol{r} \tag{S1}$$

where $\sigma \in \{\alpha, \beta\}$ is the spin index; $e_{\text{x-sr},\sigma}^{\mathrm{LDA}}$, $e_{\text{c-ss},\sigma}^{\mathrm{LDA}}$, and $e_{\text{c-os}}^{\mathrm{LDA}}$ are short-range exchange, same-spin correlation and opposite-spin correlation energy densities within local (spin) density approximation. The partition of correlation energy into same-spin and opposite-spin contributions adopts the widely-used scheme proposed by Stoll et al. (*59*). The short-range LDA exchange energy density $e_{\text{x-sr},\sigma}^{\mathrm{LDA}}$ is obtained by multiplying the LDA exchange energy density $e_{\mathrm{x},\sigma}^{\mathrm{LDA}}$ with an attenuation function

$$e_{\text{x-sr},\sigma}^{\mathrm{LDA}} = e_{\mathrm{x},\sigma}^{\mathrm{LDA}} \left[1 - \frac{2}{3} a_\sigma \left(2\sqrt{\pi}\mathrm{erf}\left(\frac{1}{a_\sigma}\right) - 3a_\sigma + a_\sigma^3 + (2a_\sigma - a_\sigma^3)\mathrm{exp}\left(-\frac{1}{a_\sigma^2}\right) \right) \right] \tag{S2}$$

where $a_\sigma = \omega / k_{\mathrm{F}\sigma}$ with $k_{\mathrm{F}\sigma} = (3\pi^2\rho)^{1/3}$ being the Fermi wave vector and ω being the range-separation parameter.

$F_{\mathrm{x},\sigma}$, $F_{\text{c-ss},\sigma}$ and $F_{\text{c-os}}$ are the exchange, same-spin correlation and opposite-spin correlation enhancement factors that depends on reduced density gradient and kinetic energy density

$$F_{\mathrm{x},\sigma} = F_{\mathrm{x},\sigma}(x_\sigma^2, w_\sigma),\ F_{\text{c-ss},\sigma} = F_{\text{c-ss},\sigma}(x_\sigma^2, w_\sigma),\ F_{\text{c-os}} = F_{\text{c-os}}(x_{\mathrm{ave}}^2, w_{\mathrm{ave}}) \tag{S3}$$

where $x_\sigma = \frac{|\boldsymbol{\nabla}\rho_\sigma|}{\rho_\sigma^{4/3}}$ denotes the reduced density gradient. w_σ is an auxiliary quantity that depends on kinetic energy density $\tau_\sigma = \frac{1}{2}\sum_i^{\mathrm{occ}} |\boldsymbol{\nabla}\psi_{i\sigma}|^2$, with ψ's being Kohn-Sham orbitals and the summation runs over occupied Kohn-Sham orbitals. In particular, $w_\sigma = (t_\sigma - 1)/(t_\sigma + 1)$, with $t_\sigma = \tau_\sigma^{\mathrm{HEG}}/\tau_\sigma$ where $\tau_\sigma^{\mathrm{HEG}} = \frac{3}{10}(6\pi^2)^{2/3}\rho_\sigma^{5/3}$ is the kinetic energy density of homogenous

electron gas (HEG). The opposite-spin correlation enhancement factor $F_{\text{c-ss},\sigma}$ depends on spin-averaged version of x^2 and w, defined as $x^2_{\text{ave}} = \frac{1}{2}(x^2_\alpha + x^2_\beta)$ and $w_{\text{ave}} = (t_{\text{ave}} - 1)/(t_{\text{ave}} + 1)$ with $t_{\text{ave}} = \frac{1}{2}(t_\alpha + t_\beta)$. We note that the form of input features for enhancement factors defined here are widely-used in B97-inspired functional forms.

The nonlocal part of the exchange-correlation functional contains the short-range exact-exchange $E^{\text{exact}}_{\text{x-sr}}$, long-range exact exchange $E^{\text{exact}}_{\text{x-lr}}$ and VV10 nonlocal correlation $E^{\text{VV10}}_{\text{c}}$. The short-range and long-range exact exchange assume the following form

$$E^{\text{exact}}_{\text{x-sr}}[\rho] = -\frac{c_{\text{x}}}{2}\sum_\sigma \sum_{i,j}^{\text{occ}} \int\int \psi^*_{i\sigma}(\boldsymbol{r}_1)\psi^*_{j\sigma}(\boldsymbol{r}_2)\frac{\text{erfc}(\omega r)}{r}\psi_{j\sigma}(\boldsymbol{r}_1)\psi_{i\sigma}(\boldsymbol{r}_2)d\boldsymbol{r}_1 d\boldsymbol{r}_2 \qquad \text{(S4)}$$

$$E^{\text{exact}}_{\text{x-lr}}[\rho] = -\frac{1}{2}\sum_\sigma \sum_{i,j}^{\text{occ}} \int\int \psi^*_{i\sigma}(\boldsymbol{r}_1)\psi^*_{j\sigma}(\boldsymbol{r}_2)\frac{\text{erf}(\omega r)}{r}\psi_{j\sigma}(\boldsymbol{r}_1)\psi_{i\sigma}(\boldsymbol{r}_2)d\boldsymbol{r}_1 d\boldsymbol{r}_2 \qquad \text{(S5)}$$

where $r = |\boldsymbol{r}_1 - \boldsymbol{r}_2|$ and ω is a range-separation parameter controlling the characteristic length scale for range separation. Note that there is a prefactor c_{x} controlling the amount of short-range exact exchange used in the functional form. The exchange functional used in this work thus behaves as purely exact exchange in long range and a mixture of semilocal and exact exchange in short range. The VV10 nonlocal correlation $E^{\text{VV10}}_{\text{c}}$ assumes the form

$$E^{\text{VV10}}_{\text{c}}[\rho] = \int \rho(\boldsymbol{r}_1)\left[\frac{1}{32}\left[\frac{3}{b^2}\right]^{3/4} + \frac{1}{2}\int \rho(\boldsymbol{r}_2)\Phi(\boldsymbol{r}_1, \boldsymbol{r}_2; b, C)d\boldsymbol{r}_2\right]d\boldsymbol{r}_1 \qquad \text{(S6)}$$

where integration kernel Φ depends on two empirical parameters b and C (see Ref. (*76*) for expression). We keep all the empirical parameters in nonlocal terms to be identical to those in ωB97M-V, namely $\omega = 0.3$, $c_{\text{x}} = 0.15$, $b = 6$ and $C = 0.01$.

2 Evolution of symbolic functional forms

The simplified mathematical forms of functional forms shown in Fig. 4 of the main text is shown below. c's and γ are parameters. The same symbol (e.g. c_0) in different enhancement factors of the same functional represent different parameters. See Table S1 for numerical values for the parameters in the GAS22 functional.

GAS22-a (ωB97M-V):

$$F_{\text{x}} = c_0 + c_1 w + \frac{c_2 \gamma x^2}{1+\gamma x^2}$$

$$F_{\text{c-ss}} = c_0 + c_1 w + c_2 w^2 + \frac{c_3 \gamma^4 x^8}{(1+\gamma x^2)^4} + \frac{c_4 \gamma^3 w^4 x^6}{(1+\gamma x^2)^3}$$

$$F_{\text{c-os}} = c_0 + c_1 w + c_2 w^2 + c_3 w^6 + \frac{c_4 \gamma w^2 x^2}{1+\gamma x^2} + \frac{c_5 \gamma w^6 x^2}{1+\gamma x^2}$$

GAS22-b:

$$F_{\text{x}} = c_0 + c_1 w + \frac{c_2 \gamma x^2}{1+\gamma x^2}$$

$$F_{\text{c-ss}} = c_1 w + c_2 w^2 + \frac{c_3 \gamma^6 w^4 x^{12}}{(1+\gamma x^2)^6} + \frac{c_4 \gamma^4 x^8}{(1+\gamma x^2)^4} + \frac{\gamma x^2}{1+\gamma x^2}$$

$$F_{\text{c-os}} = c_0 + c_2 w^2 + c_3 w^6 + \frac{c_4 \gamma w^6 x^2}{1+\gamma x^2} + \frac{c_5 \gamma w^2 x^2}{1+\gamma x^2}$$

GAS22-c:

$$F_{\text{x}} = c_0 + c_1 w + \frac{c_2 \gamma x^2}{1+\gamma x^2}$$

$$F_{\text{c-ss}} = c_1 w + c_2 w^2 + \frac{c_3 \gamma^6 w^4 x^{12}}{(1+\gamma x^2)^6} + \frac{c_4 \gamma^6 x^{12}}{(1+\gamma x^2)^6} + \frac{\gamma x^2}{1+\gamma x^2}$$

$$F_{\text{c-os}} = c_0 + c_2 w^2 + c_3 w^6 + c_4 w^6 \sqrt[3]{x^2} + c_5 w^2 \sqrt[3]{x^2}$$

GAS22:

$$F_{\text{x}} = c_0 + c_1 w + \frac{c_2 \gamma x^2}{1+\gamma x^2}$$

$$F_{\text{c-ss}} = c_1 w + c_2 w^2 + \frac{c_3 \gamma^6 w^4 x^{12}}{(1+\gamma x^2)^6} + \frac{c_4 \gamma^6 x^{12}}{(1+\gamma x^2)^6} + \frac{\gamma x^2}{1+\gamma x^2}$$

$$F_{\text{c-os}} = c_0 + c_2 w^2 + c_3 w^6 + c_4 w^6 \sqrt[3]{x^2} + c_5 w^2 \sqrt[3]{x^2}$$

	F_{x}
c_0	0.862139736374172
c_1	0.317533683085033
c_2	0.936993691972698
γ	0.003840616724010807
	$F_{\text{c-ss}}$
c_1	-4.10753796482853
c_2	-5.24218990333846
c_3	7.5380689617542
c_4	-1.76643208454076
γ	0.46914023462026644
	$F_{\text{c-os}}$
c_0	0.805124374375355
c_2	7.98909430970845
c_3	-7.54815900595292
c_4	2.00093961824784
c_5	-1.76098915061634

Table S1: Parameters in the GAS22 functional.

3 Symbolic representations of density functionals

As stated in the Table 1 of the main text, the instructions used in this work include 3 categories: arithmetic operations, power operations and building blocks from existing functionals. For the category of building blocks of existing functionals, we considered a few additional instructions in addition to the $\gamma x/(1+\gamma x)$ presented in Table 1, including PBE exchange enhancement factor $F_{\text{x}}^{\text{PBE}}$ (*63*), RPBE exchange enhancement factor $F_{\text{x}}^{\text{RPBE}}$ (*77*), B88 exchange enhancement factor $F_{\text{x}}^{\text{B88}}$ (*78*) and PBE correlation energy functional $E_{\text{c}}^{\text{PBE}}$ (*63*).

We design the probability such that similar instructions receive identical probabilities and probabilities distribute evenly among different types of instructions. For the 5 arithmetic operations, each operation receive a probability of 0.06; for the 6 power instructions, each receive a probability of 0.05; u transform receive a probability of 0.1, and the other 4 building block receive a 0.075 each.

Symbolic representation of ωB97M-V:

Algorithm 1: $F_{\text{x}}^{\omega\text{B97M-V}}$

Features: w, x^2
Variables: F, v_0, v_1
Parameters: γ, c_{00}, c_{10}, c_{01}
Instructions:

$v_0 = \gamma x^2/(1+\gamma x^2)$
$F = c_{00} + F$
$v_1 = c_{10} \times w$
$F = F + v_1$
$v_1 = c_{01} \times v_0$
$F = F + v_1$
return F

Algorithm 2: $F_{\text{c-ss}}^{\omega\text{B97M-V}}$

Features: w, x^2
Variables: F, v_0, v_1, v_2, v_3
Parameters: γ, c_{00}, c_{10}, c_{20}, c_{43}, c_{04}
Instructions:

$v_0 = \gamma x^2/(1 + \gamma x^2)$
$F = c_{00} + F$
$F+ = c_{10} \times w$
$v_1 = w^2$
$F+ = c_{20} \times v_1$
$v_1 = w^4$
$v_2 = v_0^3$
$v_3 = v_3 \times v_2$
$F+ = c_{43} \times v_3$
$v_2 = v_0^4$
$F+ = c_{04} \times v_2$
return F

Algorithm 3: $F_{\text{c-os}}^{\omega\text{B97M-V}}$

Features: w, x^2
Variables: F, v_0, v_1, v_2, v_3
Parameters: γ, c_{00}, c_{10}, c_{20}, c_{21}, c_{60}, c_{61}
Instructions:

$v_0 = \gamma x^2/(1 + \gamma x^2)$
$F = c_{00} + F$
$F+ = c_{10} \times w$
$v_1 = w^2$
$F+ = c_{20} \times v_1$
$v_3 = v_1 \times v_0$
$F+ = c_{21} \times v_1$
$v_1 = w^6$
$F+ = c_{60} \times v_1$
$v_3 = v_1 \times v_0$
$F+ = c_{61} \times v_3$
return F

4 Enhancement factors of symbolic functionals

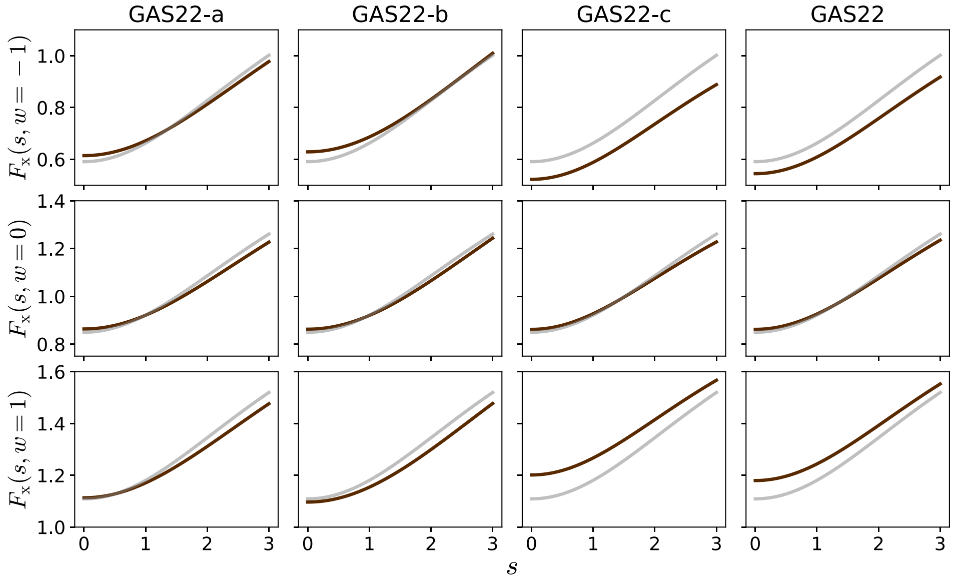

Figure S1: **Exchange enhancement factors $F_\mathbf{x}$ for functional forms in main text Fig. 4.** For reference, the enhancement factor for the ωB97M-V functional is plotted in grey.

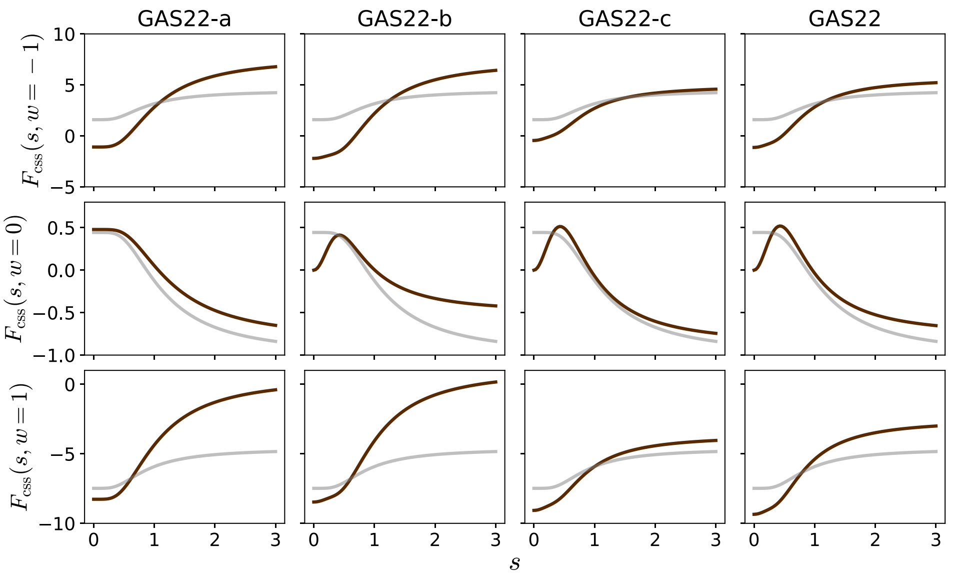

Figure S2: **Same-spin correlation enhancement factors $F_{\textbf{c-ss}}$ for functional forms in main text Fig. 4.** For reference, the enhancement factor for the ωB97M-V functional is plotted in grey.

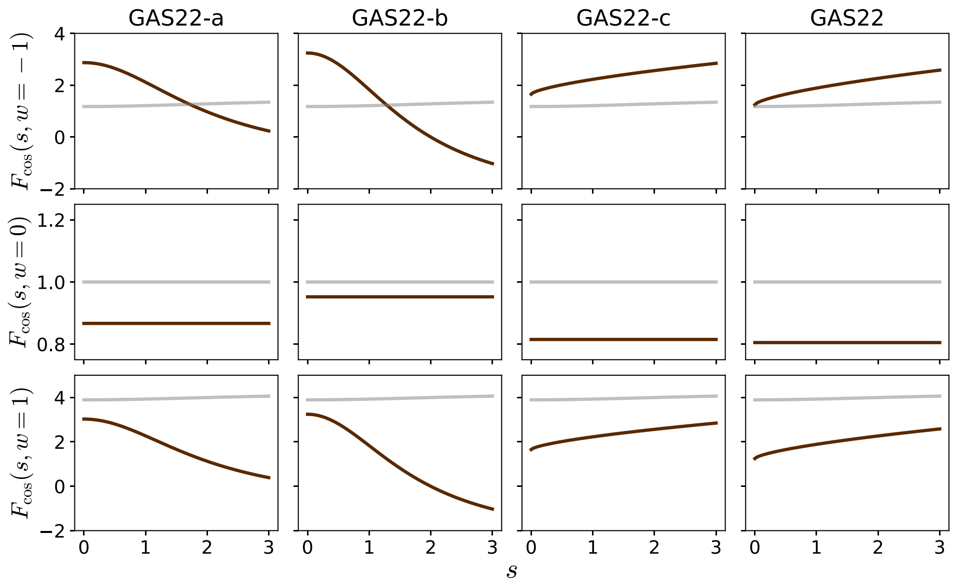

Figure S3: **Opposite-spin correlation enhancement factors $F_{\textbf{c-os}}$ for functional forms in main text Fig. 4.** For reference, the enhancement factor for the ωB97M-V functional is plotted in grey.

5 Random search studies starting from ωB97M-V

In main text Fig. 4 we presented regularized evolution calculations starting from the ωB97M-V functional. For comparison, in Fig. S4 we report random search calculations (dash lines). The random search studies are performed with identical set up as regularized evolution experiments, except that the tournament size is set to 1. Therefore, in each iteration of random search experiment, the parent functional used for mutation is randomly selected from the population without referring to the fitness of functional forms. Compared to regularized evolution calculations, random search is found to be ineffective in traversing the search space and generating better functional forms than existing forms.

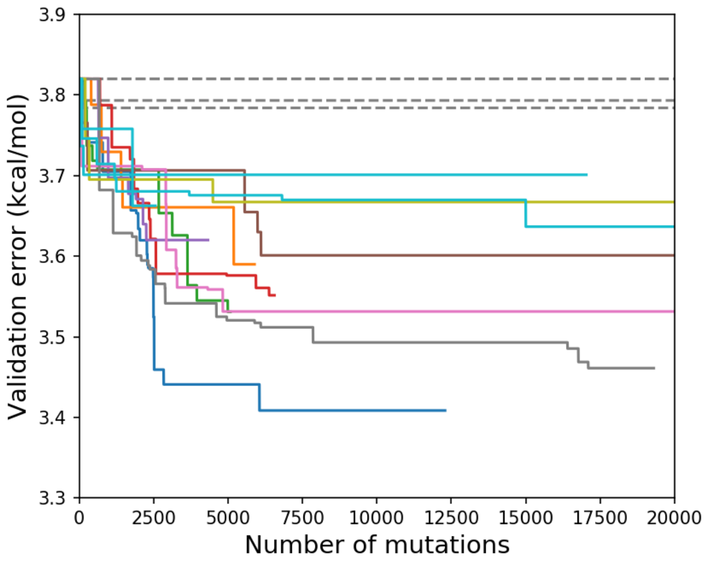

Figure S4: **Validation error of symbolic functionals generated by random search and regularized evolution experiments starting from the ωB97M-V functional.** Random search and regularized evolution results are shown with dashed (solid) lines, with different lines represent independent experiments. The reference values in MGCDB84 database are used as targets for training and evaluation of functionals.

Here we make some additional remark on the starting point (termed GAS22-a in the main

text) of the regularized evolution and random search studies. GAS22-a has identical symbolic form as ωB97M-V, but with all parameters (including γ's) optimized on the training set as done for all the symbolic functional forms generated in this work. In the original work that created the ωB97M-V functional, the nonlinear parameters γ's are not optimized and only linear parameters are optimized. Thus GAS22-a is a different functional as ωB97M-V and have different training/validation/test errors: 2.97/3.82/4.47 kcal/mol.

6 Software design

In Fig. S5 we present the high-level software design of the distributed regularized evolution program. The program consists of a population server, a population database, a fingerprint server for functional equivalence checking and a number of workers for training and evaluating functional forms. The training of a functional form is performed with the CMA-ES algorithm, which require to compute the the training error on tens of thousands of sets of different parameters. Such calculations are efficiently performed by porting the calculation of training errors to GPU processors through just-in-time compilation.

As briefly mentioned in the main text, one functional form may have multiple equivalent symbolic representations. For the purpose the functional equivalence checking, we define equivalent forms as forms that evaluates to the same value given same parameters and features, and we do not consider more complicated forms of equivalence such as the those requiring a mapping of parameters (e.g. the equivalence of B97 exchange functional and the symbolic functional obtained in main text).

To check for equivalent functional forms and avoid duplicated computations, each functional is assigned a *fingerprint*. The fingerprint is evaluated by computing the functional values using a set of features and parameters that are randomly chosen but kept consistent during the entire program. The functional values are then hashed and the hash value serves as the functional

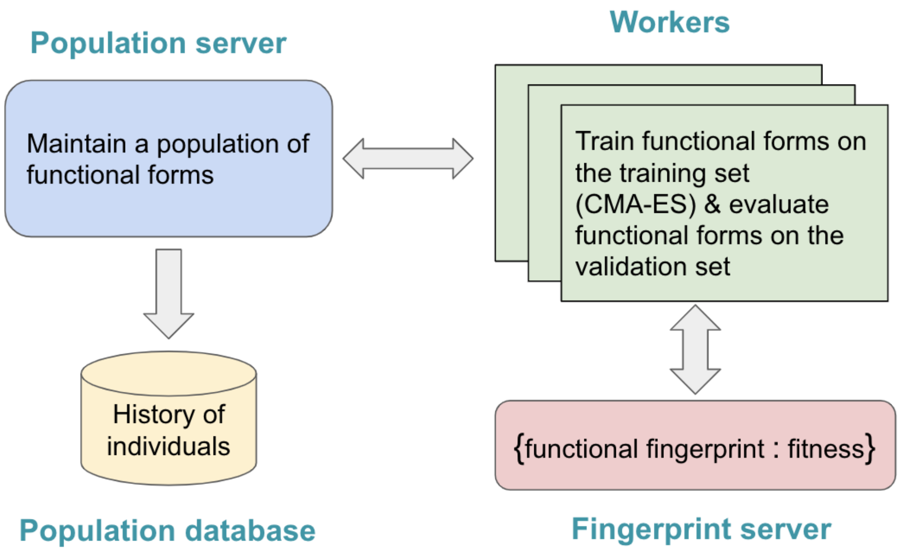

Figure S5: **Distributed design of symbolic regression software program.** The program consists of a population server, a population database, a fingerprint server for functional equivalence checking and a number of workers for training and evaluating functional forms. The regularized evolution process is performed on the population server, and all child functionals are sent to workers for training and evaluation. The workers will first check if equivalence forms are already explored. If equivalence forms are explored before, the worker will directly send the cached fitness value in fingerprint server to the population server.

fingerprint. The fingerprint is identical across all equivalent functional forms because they all evaluates to the same values with same parameters and features. All fingerprints and fitness values of explored functionals are cached during the regularized evolution calculations. Every time a new functional form is generated from mutation, its fingerprint will be evaluated to check if equivalent forms have already been explored. If equivalent forms are explored before, the cached fitness values are used without re-training the functional form.

7 Colab notebook demonstration of GAS22 in self-consistent calculations

We provide an example Colab notebook to demonstrate self-consistent DFT calculations with GAS22 functional at https://colab.research.google.com/github/google-research/google-research/blob/master/symbolic_functionals/colab/run_GAS22.ipynb.

REFERENCES AND NOTES

1. P. Hohenberg, W. Kohn, Inhomogeneous electron gas. *Phys. Rev.* **136**, B864 (1964).

2. R. M. Martin, *Electronic Structure: Basic Theory and Practical Methods* (Cambridge Univ. Press, 2020).

3. R. O. Jones, Density functional theory: Its origins, rise to prominence, and future. *Rev. Mod. Phys.* **87**, 897–923 (2015).

4. N. Mardirossian, Y. Wang, D. A. Pearlman, G. K. Chan, T. Shiozaki, Novel algorithms and high-performance cloud computing enable efficient fully quantum mechanical protein-ligand scoring. arXiv:2004.08725 [physics.chem-ph] (18 April 2020).

5. J. K. Nørskov, F. Abild-Pedersen, F. Studt, T. Bligaard, Density functional theory in surface chemistry and catalysis. *Proc. Natl. Acad. Sci.* **108**, 937–943 (2011).

6. G. Galli, The long and winding road: Predicting materials properties through theory and computation, in *Handbook of Materials Modeling: Methods: Theory and Modeling* (2020), pp. 37–48.

7. W. Kohn, L. J. Sham, Self-consistent equations including exchange and correlation effects. *Phys. Rev.* **140**, A1133–A1138 (1965).

8. A. D. Becke, Perspective: Fifty years of density-functional theory in chemical physics. *J. Chem. Phys.* **140**, 18A301 (2014).

9. H. S. Yu, S. L. Li, D. G. Truhlar, Perspective: Kohn-sham density functional theory descending a staircase. *J. Chem. Phys.*, 130901 (2016).

10. L. Li, K. Burke, Recent developments in density functional approximations, in *Handbook of Materials Modeling: Methods: Theory and Modeling* (2020), pp. 213–226.

11. N. Mardirossian, M. Head-Gordon, Thirty years of density functional theory in computational chemistry: An overview and extensive assessment of 200 density functionals, *Mol. Phys.* **115**, 2315–2372 (2017).

12. J.-D. Chai, M. Head-Gordon, Systematic optimization of long-range corrected hybrid density functionals. *J. Chem. Phys.* **128**, 084106 (2008).

13. N. Mardirossian, M. Head-Gordon, ωb97x-v: A 10-parameter, range-separated hybrid, generalized gradient approximation density functional with nonlocal correlation, designed by a survival-of-the-fittest strategy. *Phys. Chem. Chem. Phys.* **16**, 9904–9924 (2014).

14. N. Mardirossian, M. Head-Gordon, Mapping the genome of meta-generalized gradient approximation density functionals: The search for b97m-v, *J. Chem. Phys.* **142**, 074111 (2015).

15. N. Mardirossian, M. Head-Gordon, ωb97m-v: A combinatorially optimized, range-separated hybrid, meta-gga density functional with vv10 nonlocal correlation. *J. Chem. Phys.* **144**, 214110 (2016).

16. N. Mardirossian, M. Head-Gordon, Survival of the most transferable at the top of Jacob's ladder: Defining and testing the ωb97m (2) double hybrid density functional. *J. Chem. Phys.* **148**, 241736 (2018).

17. Y. Zhao, D. G. Truhlar, Applications and validations of the minnesota density functionals. *Chem. Phys. Lett.* **502**, 1–13 (2011).

18. R. Peverati, D. G. Truhlar, Improving the accuracy of hybrid meta-gga density functionals by range separation. *J. Phys. Chem. Lett.* **2**, 2810–2817 (2011).

19. H. S. Yu, X. He, D. G. Truhlar, Mn15-l: A new local exchange-correlation functional for kohn–sham density functional theory with broad accuracy for atoms, molecules, and solids, *J. Chem. Theor. Comput.* **12**, 1280–1293 (2016).

20. Y. Wang, P. Verma, L. Zhang, Y. Li, Z. Liu, D.G. Truhlar, X. He, M06-sx screened-exchange density functional for chemistry and solid-state physics. *Proc. Natl. Acad. Sci.* **117**, 2294–2301 (2020).

21. A. D. Becke, Density-functional thermochemistry. V. Systematic optimization of exchange-correlation functionals. *J. Chem. Phys.* **107**, 8554–8560 (1997).

22. R. Peverati, D. G. Truhlar, Quest for a universal density functional: The accuracy of density functionals across a broad spectrum of databases in chemistry and physics. *Philos. Trans. R. Soc. A Math. Phys. Eng. Sci.***372**, 20120476 (2014).

23. B. Kalita, L. Li, R. J. McCarty, K. Burke, Learning to approximate density functionals. *Acc. Chem. Res.* **54**, 818–826 (2021).

24. J. C. Snyder, M. Rupp, K. Hansen, K.-R. Müller, K. Burke, Finding density functionals with machine learning. *Phys. Rev. Lett.* **108**, 253002 (2012).

25. L. Li, J. C. Snyder, I. M. Pelaschier, J. Huang, U.N. Niranjan, P. Duncan, M. Rupp, K.R. Müller, K. Burke, Understanding machine-learned density functionals. *Int. J. Quant. Chem.* **116**, 819–833 (2016).

26. L. Li, T. E. Baker, S. R. White, K. Burke, Pure density functional for strong correlation and the thermodynamic limit from machine learning. *Phys. Rev. B* **94**, 245129 (2016).

27. F. Brockherde, L. Vogt, L. Li, M. E. Tuckerman, K. Burke, K.-R. Müller, Bypassing the kohn-sham equations with machine learning. *Nat. Commun.* **8**, 872 (2017).

28. M. Bogojeski, L. Vogt-Maranto, M. E. Tuckerman, K.-R. Müller, K. Burke, Quantum chemical accuracy from density functional approximations via machine learning. *Nat. Commun.* **11**, 5223 (2020).

29. J. Seino, R. Kageyama, M. Fujinami, Y. Ikabata, H. Nakai, Semi-local machine-learned kinetic energy density functional with third-order gradients of electron density. *J. Chem. Phys.* **148**, 241705 (2018).

30. K. Ryczko, D. A. Strubbe, I. Tamblyn, Deep learning and density-functional theory. *Phys. Rev. A* **100**, 022512 (2019).

31. M. Fujinami, R. Kageyama, J. Seino, Y. Ikabata, H. Nakai, Orbital-free density functional theory calculation applying semi-local machine-learned kinetic energy density functional and kinetic potential. *Chem. Phys. Lett.* **748**, 137358 (2020).

32. R. Meyer, M. Weichselbaum, A. W. Hauser, Machine learning approaches toward orbital-free density functional theory: Simultaneous training on the kinetic energy density functional and its functional derivative. *J. Chem. Theor. Comput.* **16**, 5685–5694 (2020).

33. J. Wellendorff, K. T. Lundgaard, A. Møgelhøj, V. Petzold, D. D. Landis, J. K. Nørskov, T. Bligaard, K. W. Jacobsen, Density functionals for surface science: Exchange-correlation model development with bayesian error estimation. *Phys. Rev. B* **85**, 235149 (2012).

34. K. T. Lundgaard, J. Wellendorff, J. Voss, K. W. Jacobsen, T. Bligaard, mbeef-vdw: Robust fitting of error estimation density functionals. *Phys. Rev. B* **93**, 235162 (2016).

35. M. Gastegger, L. González, P. Marquetand, Exploring density functional subspaces with genetic algorithms. *Monatsh. Chem.* **150**, 173–182 (2019).

36. R. A. Vargas-Hernandez, Bayesian optimization for calibrating and selecting hybrid-density functional models, *J. Phys. Chem. A* **124**, 4053–4061 (2020).

37. K. Hornik, Approximation capabilities of multilayer feedforward networks. *Neural Netw.* **4**, 251–257 (1991).

38. L. Li, S. Hoyer, R. Pederson, R. Sun, E. D. Cubuk, P. Riley, K. Burke, Kohn-Sham equations as regularizer: Building prior knowledge into machine-learned physics. *Phys. Rev. Lett.* **126**, 036401 (2021).

39. M. F. Kasim, S. M. Vinko, Learning the exchange-correlation functional from nature with fully differentiable density functional theory, *Phys. Rev. Lett.* **127**, 126403 (2021).

40. S. Dick, M. Fernandez-Serra, Using differentiable programming to obtain an energy and density-optimized exchange-correlation functional. arXiv:2106.04481 [physics.chem-ph] (8 June 2021).

41. R. Nagai, R. Akashi, O. Sugino, Completing density functional theory by machine learning hidden messages from molecules. *npj Comput. Mater.* **6**, 43 (2020).

42. R. Nagai, R. Akashi, O. Sugino, Machine-learning-based exchange correlation functional with physical asymptotic constraints. *Phys. Rev. Res.* **4**, 013106 (2022).

43. Y. Chen, L. Zhang, H. Wang, W. E, Deepks: A comprehensive data-driven approach toward chemically accurate density functional theory. *J. Chem. Theor. Comput.* **17**, (2021).

44. S. Dick, M. Fernandez-Serra, Machine learning accurate exchange and correlation functionals of the electronic density. *Nat. Commun.* **11**, 3509 (2020).

45. J. Kirkpatrick, B. McMorrow, D. H. P. Turban, A. L. Gaunt, J. S. Spencer, A. G. D. G. Matthews, A. Obika, L. Thiry, M. Fortunato, D. Pfau, L.R. Castellanos, S. Petersen, A. W. R. Nelson, P. Kohli, P. Mori-Sánchez, D. Hassabis, A. J. Cohen, Pushing the frontiers of density functionals by solving the fractional electron problem, *Science* **374**, 1385–1389 (2021).

46. M. Schmidt, H. Lipson, Distilling free-form natural laws from experimental data, *Science* **324**, 81–85 (2009).

47. S.-M. Udrescu, M. Tegmark, Ai Feynman: A physics-inspired method for symbolic regression. *Sci. Adv.* **6**, eaay2631 (2020).

48. R. Ouyang, S. Curtarolo, E. Ahmetcik, M. Scheffler, L. M. Ghiringhelli, Sisso: A compressed-sensing method for identifying the best low-dimensional descriptor in an immensity of offered candidates, *Phys. Rev. Mater.* **2**, 083802 (2018).

49. C. J. Bartel, C. Sutton, B. R. Goldsmith, R. Ouyang, C. B. Musgrave, L. M. Ghiringhelli, M. Scheffler, New tolerance factor to predict the stability of perovskite oxides and halides. *Sci. Adv.* **5**, eaav0693 (2019).

50. L. Li, M. Fan, R. Singh, P. Riley, Neural-guided symbolic regression with semantic prior. arXiv:1901.07714 [cs.LG] (23 January 2019).

51. M. D. Cranmer, A. Sanchez-Gonzalez, P. Battaglia, R. Xu, K. Cranmer, D. Spergel, S. Ho, Discovering symbolic models from deep learning with inductive biases, in *Proceedings of the 34th Conference on Neural Information Processing Systems (NeurIPS 2020)*, Vancouver, Canada, 6 to 12 December 2020 (2020).

52. A. Hernandez, A. Balasubramanian, F. Yuan, S. A. Mason, T. Mueller, Fast, accurate, and transferable many-body interatomic potentials by symbolic regression. *npj Comput. Mater.* **5**, 112 (2019).

53. E. Kabliman, A. H. Kolody, J. Kronsteiner, M. Kommenda, G. Kronberger, Application of symbolic regression for constitutive modeling of plastic deformation. *Appl. Eng. Sci.* **6**, 100052 (2021).

54. Y. Wang, N. Wagner, J. M. Rondinelli, Symbolic regression in materials science. *MRS Commun.* **9**, 793–805 (2019).

55. H. Vaddireddy, A. Rasheed, A. E. Staples, O. San, Feature engineering and symbolic regression methods for detecting hidden physics from sparse sensor observation data, *Phys. Fluids* **32**, 015113 (2020).

56. F. Sun, Y. Liu, J.-X. Wang, H. Sun, Symbolic physics learner: Discovering governing equations via Monte Carlo tree search. arXiv:2205.13134 [cs.AI] (26 May 2022).

57. E. Real, A. Aggarwal, Y. Huang, Q. V. Le, Regularized evolution for image classifier architecture search. *Proc. AAAI Conf. Artif. Intell.* **33**, 4780–4789 (2019).

58. J. P. Perdew, Y. Wang, Accurate and simple analytic representation of the electron-gas correlation energy, *Phys. Rev. B* **45**, 13244–13249 (1992).

59. H. Stoll, C. Pavlidou, H. Preuß, Correlation energies in the spin-density functional formalism. *Theor. Chim. Acta* **55**, 29–41 (1980).

60. E. Real, C. Liang, D. R. So, Q. V. Le, AutoML-Zero: Evolving machine learning algorithms from scratch, in *Proceedings of 37th International Conference on Machine Learning (ICML)*, 13 to 18 July 2020 (ICML, 2020).

61. J. D. Co-Reyes, Y. Miao, D. Peng, E. Real, S. Levine, Q. V. Le, H. Lee, A. Faust, Evolving reinforcement learning algorithms. arXiv:2101.03958 [cs.LG] (8 January 2021).

62. J. Sun, B. Xiao, Y. Fang, R. Haunschild, P. Hao, A. Ruzsinszky, G.I. Csonka, G.E. Scuseria, J.P. Perdew, Density functionals that recognize covalent, metallic, and weak bonds. *Phys. Rev. Lett.* **111**, 106401 (2013).

63. J. P. Perdew, K. Burke, M. Ernzerhof, Generalized gradient approximation made simple. *Phys. Rev. Lett.* **77**, 3865–3868 (1996).

64. S. Lehtola, C. Steigemann, M. J. Oliveira, M. A. Marques, Recent developments in LIBXC—A comprehensive library of functionals for density functional theory, *SoftwareX* **7**, 1–5 (2018).

65. J. P. Perdew, K. Schmidt, Jacob's ladder of density functional approximations for the exchange-correlation energy. *AIP Conf. Proc.* **577**, 1 (2001).

66. J. Deng, W. Dong, R. Socher, L.-J. Li, K. Li, L. Fei-Fei, Imagenet: A large-scale hierarchical image database, in *Proceedings of the 2009 IEEE Conference on Computer Vision and Pattern Recognition* (IEEE, 2009), pp. 248–255.

67. R. Evans, M. Oettel, R. Roth, G. Kahl, New developments in classical density functional theory. *J. Phys. Condens. Matter* **28**, 240401 (2016).

68. S.-C. Lin, G. Martius, M. Oettel, Analytical classical density functionals from an equation learning network. *J. Chem. Phys.* **152**, 021102 (2020).

69. J. F. Méndez-Valderrama, Y. A. Kinkhabwala, J. Silver, I. Cohen, T. Arias, Density-functional fluctuation theory of crowds, *Nat. Commun.* **9**, 3538 (2018).

70. A. Davies, P. Veličković, L. Buesing, S. Blackwell, D. Zheng, N. Tomašev, R. Tanburn, P. Battaglia, C. Blundell, A. Juhász, M. Lackenby, G. Williamson, D. Hassabis, P. Kohli, Advancing mathematics by guiding human intuition with ai, *Nature* **600**, 70–74 (2021).

71. J. Bradbury, Jax: Composable transformations of python+ numpy programs (2018); http://github.com/google/jax.

72. N. Hansen, Y. Akimoto, P. Baudis, CMA-ES/pycma on Github, Zenodo, 10.5281/zenodo.2559634 (2019).

73. Q. Sun, T. C. Berkelbach, N. S. Blunt, G. H. Booth, S. Guo, Z. Li, J. Liu, J. McClain, E. R. Sayfutyarova, S. Sharma, S. Wouters, G. K.-L. Chan, PySCF: The python-based simulations of chemistry framework. *Wiley Interdiscip. Rev. Comput. Mol. Sci.* **8**, e1340 (2017).

74. D. Rappoport, F. Furche, Property-optimized gaussian basis sets for molecular response calculations, *J. Chem. Phys.* **133**, 134105 (2010).

75. P. M. Gill, B. G. Johnson, J. A. Pople, A standard grid for density functional calculations, *Chem. Phys. Lett.* **209**. 506–512 (1993).

76. O. A. Vydrov, T. Van Voorhis, Nonlocal van der waals density functional: The simpler the better. *J. Chem. Phys.* **133**, 244103 (2010).

77. B. Hammer, L. B. Hansen, J. K. Nørskov, Improved adsorption energetics within density-functional theory using revised perdew-burke-ernzerhof functionals. *Phys. Rev. B* **59**, 7413–7421 (1999).

78. A. D. Becke, Density-functional exchange-energy approximation with correct asymptotic behavior. *Phys. Rev. A* **38**, 3098–3100 (1988).
